# "Mems." for Muddled Medicals

**Published:** 1889-09-14

**Authors:** 


### "Mems." for Muddled Medicals.

The first thing a young man must do who intends to become a doctor is to pass his Preliminary Examination in General Education.

No attendance at hospital or on lectures will "count" as part of the usual curriculum until after this examination has been passed.

"Registration" is the second step to be taken. No man can be registered as a medical student who has not passed the whole of the Preliminary Arts Examination.

The easiest preliminary examinations are those of the Apothecaries' Society of London, the Faculty of Physicians and Surgeons of Glasgow, and the King's and Queen's Colleges of Physicians of Ireland. The next easiest are those of the Royal College of Surgeons of England, the Royal College of Physicians of London, and the Royal College of Physicians and Surgeons, Edinburgh.

For the degree of M.B., which can only be granted by a university, the preliminary examinations are both more extensive and more difficult than for any of the college diplomas. The London University holds a special Matriculation Examination and a Preliminary Scientific. The Edinburgh University and the other Scotch universities also hold special Preliminary Examinations. These latter take place in October and March. Those intending to take the M.B. degree at Oxford or Cambridge must, at the same time or previously, also take the B.A.

Most "Qualifying Boards" require four full years, or *Anni Medici*, to have been spent in study before they grant degrees or diplomas.

An *Annus Medicus* consists of two complete courses of lectures of 100 each, given by duly recognised lecturers. Less than this does not count as an *Annus Medicus*.

The London University requires five years to have been spent in study before granting the degree of M.B. At Edinburgh, and some other Scotch universities, it is a common practice to take five years, although the curriculum can be completed in four.

After passing the Preliminary Examination, it is usual to study two full years, and then to take what is called "The First College" Examination in London. At the end of two years more the "Second College" or "Final" is taken. At the Scotch universities there are three examinations after the Preliminary—viz., the First Professional, the Second Professional, and the Final or M.B. Examination. The Victoria University at Manchester follows similar rules. The Universities of Durham and St. Andrews are chiefly used by medical men who have been for some years in practice.

The fees for hospital practice and systematic lectures vary a good deal. They are low at some of the English provincial towns, and also at Anderson's College, Glasgow, and at the Edinburgh College of Surgeons. The hospitals in London have no uniformity in this respect. Guy's, St. Thomas's, and St. Bartholomew's are high, whilst the London Hospital in Whitechapel-road is low. The Scotch universities also vary, Edinburgh exacting the highest fees and Aberdeen the lowest. The total fees for the whole curriculum range between £100 and £200. This is of course exclusive of books and living expenses.

Most of the subjects of medical study are quite new to students. It is therefore very necessary to commence attendance and work on the very first day of the session, and to attend with unbroken regularity to the last. It is of the utmost importance also to keep up private reading, and to be always abreast of the lecturer or even a little in advance. The observance of this rule would convert many "plucks" into "passes."

Food, fresh air, and books are indispensable requisites of medical student life. He who is likely to be deficient in any of these had better postpone his medical studies for a year or two, or even give up the idea of medicine altogether.

In reading it is better to read well than to read much. No young man should read later than ten o'clock at night. It is better to leave off at nine, and to have half an hour's run before going to bed.

Anatomy is best learnt in the dissecting-room, physiology in the laboratory, and chemistry also in the laboratory. The facilities for practical study are excellent at some medical schools, at others they are disgraceful.

The medical and surgical wards of the hospital are the places for studying diseases and accidents. Every medical student should be a "dresser" and "clinical clerk" for as many months and years as he has opportunity. Nothing can supply the want of hospital experience.

The use of books is to teach the knowledge of "things." It does not matter how well any particular book is known if the "things" are not understood and mastered. When the book fails to teach, the demonstrator, or the professor, or a quiet fellow-student should invariably be consulted.

